# Seroprevalence of SARS-CoV-2 and Infection Fatality Ratio, Orleans and Jefferson Parishes, Louisiana, USA, May 2020

**DOI:** 10.3201/eid2611.203029

**Published:** 2020-11

**Authors:** Amy K. Feehan, Daniel Fort, Julia Garcia-Diaz, Eboni G. Price-Haywood, Cruz Velasco, Eric Sapp, Dawn Pevey, Leonardo Seoane

**Affiliations:** Ochsner Clinic Foundation, New Orleans, Louisiana, USA (A.K. Feehan, D. Fort, J. Garcia-Diaz, E.G. Price-Haywood, C. Velasco, D. Pevey, L. Seoane);; University of Queensland Ochsner Clinical School, New Orleans (A.K. Feehan, J. Garcia-Diaz, E.G. Price-Haywood, L. Seoane);; Public Democracy, Arlington, Virginia, USA (E. Sapp);; Louisiana State University Health Science Center–Shreveport, Shreveport, Louisiana, USA (L. Seoane)

**Keywords:** COVID-19, coronavirus disease, SARS-CoV-2, severe acute respiratory syndrome coronavirus 2, viruses, respiratory infections, zoonoses, cross-sectional studies, healthcare disparities, convalescence, prevalence, seroepidemiologic studies, New Orleans, Louisiana, United States

## Abstract

Using a novel recruitment method and paired molecular and antibody testing for severe acute respiratory syndrome coronavirus 2 infection, we determined seroprevalence in a racially diverse municipality in Louisiana, USA. Infections were highly variable by ZIP code and differed by race/ethnicity. Overall census-weighted seroprevalence was 6.9%, and the calculated infection fatality ratio was 1.63%.

Seroprevalence studies around the world have estimated the spread of severe acute respiratory syndrome coronavirus 2 (SARS-CoV-2) to range from 1.79% (*1*) in Boise, Idaho, USA, to 25% in Breves, Brazil (P. Hallal, unpub. data, https://doi.org/10.1101/2020.05.30.20117531). Coronavirus disease (COVID-19) has also been reported to disproportionately affect Black patients, but we do not know the infection fatality ratio (IFR), which requires knowing how many persons are at risk (i.e., infected). We estimated SARS-CoV-2 infections in Orleans and Jefferson Parishes, Louisiana, USA, and determined the COVID-19–related IFR by race.

The protocol was approved by the Ochsner Clinic Foundation Institutional Review Board (New Orleans, LA, USA) and designed to enroll and test up to 3,000 persons at 10 sites during May 9–15, 2020. To recruit a representative sample for this high-throughput method, a novel 2-step system developed by Public Democracy (https://www.publicdemocracy.io) considered >50 characteristics, including social determinants of health and US Census population data, to establish a pool of potential participants reflective of the demographics of the parishes, from which a randomized subset of 150,000 was selected. Of these, >25,000 volunteers were recruited through dynamic, cross-device digital advertisements, supplemented by television advertisements and a call-in number to register (Appendix). This volunteer pool was stratified by the same attributes and then randomly issued a text message inviting them to private testing locations. Invitations were adjusted daily on the basis of response rates to achieve a representative sample. Volunteers checked in with a digital code to discourage unsolicited walk-ins. We did not turn uninvited persons away but excluded them from analysis if they did not fit criteria. Housemates of participants (n = 234) or persons from ineligible ZIP codes (n = 34) were excluded. Six people withdrew consent. All study materials were created in English, Spanish, and Vietnamese. Participants were offered free transportation if needed. Verbal consent was electronically documented, and participants were asked a short list of questions followed by a blood draw and nasopharyngeal swab.

Tests approved by the US Food and Drug Administration’s Emergency Use Authorization were used. Real-time reverse transcription PCR tests of nasopharyngeal swabs were performed on the Abbott *m*2000 Real*Time* System (Abbott, https://www.abbott.com) and qualitative IgG blood tests on the ARCHITECT *i*2000*SR* (Abbott). The IgG test meets criteria described by the Centers for Disease Control and Prevention as yielding high positive predictive value, which was validated by a laboratory at Ochsner Health and others (*1*,*2*). Study participants for whom either or both tests were positive were considered to be infected with SARS-CoV-2. 

US Census values, weighted by race and parish of residence, were divided by the total sample for exposure (a PCR-positive test, an IgG-positive test, or both), point prevalence (PCR-positive only), and seroprevalence (IgG-positive tests regardless of PCR test result). The positive-testing population included persons with early-stage infections (PCR-positive only) and persons recovering (PCR-positive and IgG-positive) and recovered (IgG-positive only). Early-stage infections were excluded from IFR estimation because their outcomes would not yet be registered as deaths. Therefore, weighted seroprevalence was used to calculate persons presumed to be recovered (*3*). IFR was calculated by dividing cumulative deaths by race (*4*) by the number of persons presumed to be recovered. Methodology and symptoms observed have been described elsewhere (A. Feehan, unpub. data, https://ssrn.com/abstract=3633166).

Among the 2,640 persons in the sample, 63.5% were female and 60.9% were White; average age was 50.6 years, and average household size was 2.55 persons. Among the 183 participants who tested positive, 49% were Black. The unadjusted exposure rate of SARS-CoV-2 in the sample population was 6.9% (7.8%, census-weighted); 0.9% were positive for active viral shedding but had no detectable antibody. By race, seroprevalence was highest (9.8%) in Black participants, followed by multiracial (7.1%), Asian (5.5%), and White (4.5%) participants. Hispanic participants had 5.3% seroprevalence. We multiplied 2018 population estimates by weighted seroprevalence to generate the number of persons presumed to be recovered (Table). Reported deaths (*4*) were divided by number of persons presumed to be recovered plus deaths to calculate the IFR, which was 1.61% overall. The IFR was statistically similar for White (1.55%), Black (1.69%), and multiracial (1.38%) persons but was significantly lower for Asian persons (0.61%). No COVID-19–related data on Hispanic persons were collected by the Louisiana Department of Public Health during the study period.

**Table:** Prevalence of SARS-CoV-2 infection and COVID-19–related IFR after 7 weeks of an active stay-at-home order, by race/ethnicity, 10 sites in Orleans and Jefferson Parishes, Louisiana, USA, May 9–15, 2020*

Value	Total	White	Black	Asian	Native American	Pacific Islander	Multiracial or other	Hispanic†
Positive, no./total no. (%)	183/2,640 (100)	79/1,607 (60.9)	90/828 (31.4)	9/130 (4.9)	0/14 (0.5)	0/3 (0.1)	5/58 (2.2)	18/293 (11.1)
Orleans/Jefferson Parish residents, no. (%)	825,057 (100)	419,800 (50.8)	356,925 (43.2)	29,740 (3.6)	4,088 (0.5)	495 (0.1)	14,009 (1.7)	86,289 (10.5)
Unadjusted exposure‡	6.9 (6.0–8.0)	4.9 (3.9–6.1)	10.9 (8.8–13.2)	6.9 (3.2–12.7)	0	0	8.6 (2.9–19.0)	6.1 (3.7–9.5)
Weighted exposure§	7.8 (7.8–7.9)	5.9 (5.8–5.9)	10.3 (10.2–10.4)	6.4 (6.1–6.7)	0	0	9.4 (9.0–10.0)	7.5 (7.3– 7.7)
Weighted point prevalence¶	1.0 (0.6–1.3)	1.3 (0.8–1.9)	0.5 (0–1.0)	0.9 (0–2.6)	0	0	2.2 (0–5.9)	2.2 (0.5–3.8)
Weighted seroprevalence#	6.9 (6.8–6.9)	4.5 (4.4–4.6)	9.8 (9.7–9.9)	5.5 (5.2–5.7)	0	0	7.1 (6.7–7.6)	5.3 (5.2–5.5)
No. presumed recovered**	56,578	18,975	34,973	1,629	–	–	1,001	4,582
No. deaths as of May 16, 2020	925	299	600	10	0	2	14	Unknown
IFR††	1.61 (1.5–1.7)	1.55 (1.4–1.7)	1.69 (1.6–1.8)	0.61 (0.3–1.1)‡‡	–	–	1.38 (0.8–2.3)	–

*Values are % (95% CI) except as indicated. The 2018 population estimates and deaths by race reported by the Louisiana Department of Public Health (*4*). Deaths are deemed to be COVID-19–related and have an associated confirmed PCR-positive test. Probable COVID-19 deaths without a positive PCR test were not included in these counts. By May 16, a total of 13,666 state-aggregated, confirmed cases had been reported in both parishes. COVID-19, coronavirus disease; IFR, infection fatality ratio; SARS-CoV-2, severe acute respiratory syndrome coronavirus 2; –, calculated value would be unreliable given low sample. †Hispanic ethnicity is a separate analysis and numbers were not subtracted from race. Hispanic deaths were not being reported as of May 16, 2020 (*4*). ‡Percentage of the sample with a PCR-positive test, an IgG-positive test, or both. §Census-weighted percentage of a PCR-positive test, an IgG-positive test, or both calculated to match 2018 racial demographics by parish and then combined. ¶Census-weighted percentage of PCR-positive and IgG-postive tests calculated to match 2018 racial demographics by parish and then combined. #Census-weighted percentage of IgG-positive tests calculated to match 2018 racial demographics by Parish and then combined. **Number of residents multiplied by weighted seroprevalence (IgG-positive tests). ††IFR equals the number of deaths per number of persons presumed recovered from SARS-CoV-2 infection plus deaths. ‡‡Significantly lower than White (p = 0.0034), Black (p = 0.0013), and multiracial or other (p = 0.0467) persons.

The prevalence of viral shedding (PCR-positive) and overall SARS-CoV-2 exposure (PCR-positive, IgG-positive, or both) were listed and mapped by ZIP code across the 2 parishes (Figure). Prevalence was highly variable across the map and in some areas exceeded 20%.

**Figure:**
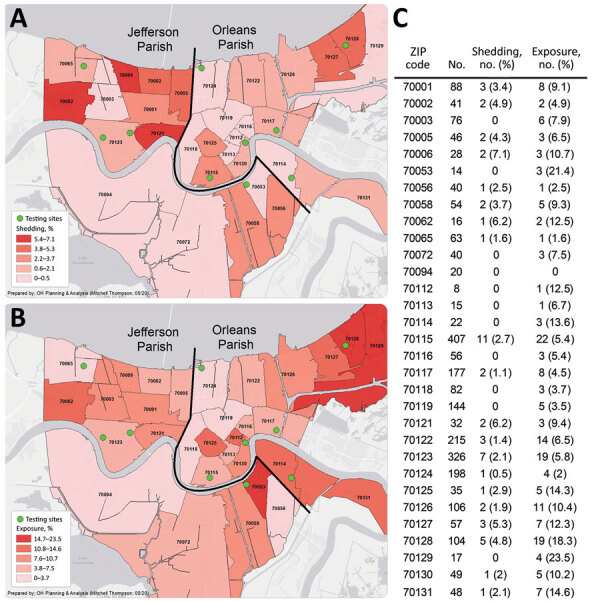
Heat maps of current and past severe acute respiratory syndrome coronavirus 2 infections after 7 weeks of an active stay-at-home order, 10 sites in Orleans and Jefferson Parishes, Louisiana, USA, May 9–15, 2020. A) Viral shedding, as indicated by PCR-positive test. B) Exposure to virus, as indicated by PCR-positive test, IgG-positive test, or both. C) Number and percentage of persons who were tested in each ZIP code, who were shedding virus (PCR-positive), and who had past or current infection (having a PCR-positive test, IgG-positive test, or both).

Prevalence studies help to understand infection spread, especially when testing resources are limited. Our study found the overall SARS-CoV-2 exposure rate in this area to be 7.8% and confirmed a recent report of overrepresentation of Black persons with COVID-19 in the New Orleans area (*5*). Multiracial, Hispanic, and Asian persons also had higher seroprevalence than White persons. The overall IFR was 1.63%, which is higher than IFRs found in other seroprevalence studies (0.5%–1.2%) (*6*; M. Emmenegger, unpub. data, https://doi.org/10.1101/2020.05.31.20118554; P. Hallal, unpub. data, https://doi.org/10.1101/2020.05.30.20117531). The similar IFR among most racial groups indicates that viral spread at least partially explains the increased number of deaths among minority populations.

Appendix Additional information about seroprevalence of SARS-CoV-2 and infection fatality ratio, Orleans and Jefferson Parishes, Louisiana, USA, May 2020.
