# Supplementary material for: Seroprevalence of SARS-CoV-2 and Infection Fatality Ratio, Orleans and Jefferson Parishes, Louisiana, USA, May 2020
Source: Emerg Infect Dis. 2020 Nov;26(11):2765–8. doi: 10.3201/eid2611.203029 (PMC7588526; doi:10.3201/eid2611.203029)
Supplement: Appendix — Additional information about seroprevalence of SARS-CoV-2 and infection fatality ratio, Orleans and Jefferson Parishes, Louisiana, USA, May 2020. [file 20-3029-Techapp-s1.pdf]

Article DOI: https://doi.org/10.3201/eid2611.203029

Seroprevalence of SARS-CoV-2 and Infection Fatality Ratio, Orleans and Jefferson Parishes, Louisiana, USA, May 2020

Appendix

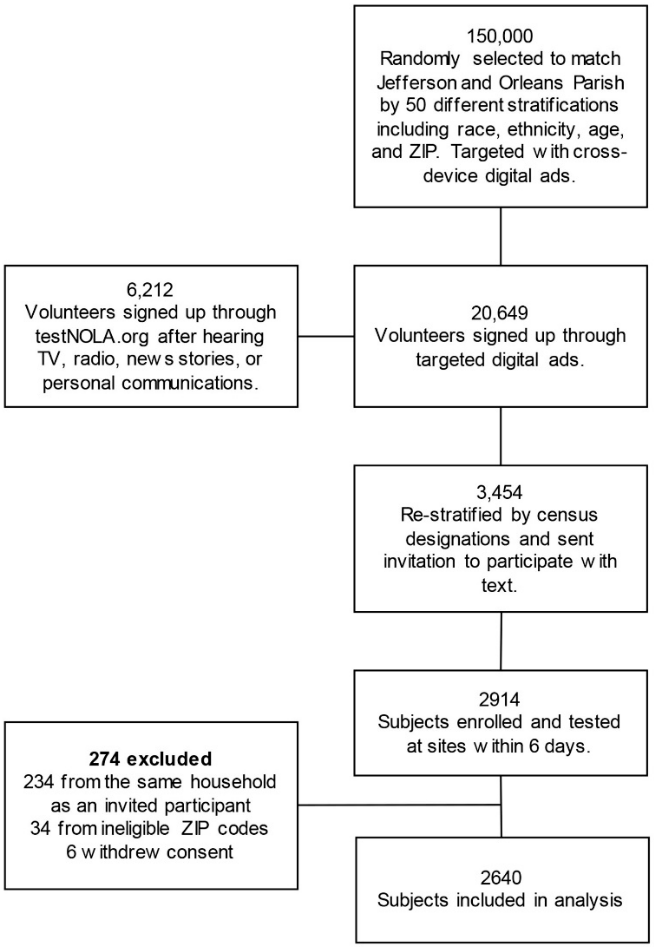

Appendix Figure. Flow diagram of recruitment efforts. From 150,000 targeted individuals, over 25,000 volunteers signed up to participate in the study. From the pool of targeted individuals (not self-selected from testNOLA.org), 3,454 were selected and invited to participate. Of those, 2,914 showed up to sites and were successfully enrolled and tested. 274 were excluded for the reasons listed and 2,640 were included in this analysis.
